# Evolution of Pollution Levels from COVID-19 Lockdown to Post-Lockdown over India

**DOI:** 10.3390/toxics10110653

**Published:** 2022-10-29

**Authors:** Bhishma Tyagi, Naresh Krishna Vissa, Sachin D. Ghude

**Affiliations:** 1 Department of Earth and Atmospheric Sciences, National Institute of Technology Rourkela, Rourkela 769008, India; 2 Indian Institute of Tropical Meteorology Pune, Pune 411008, India

**Keywords:** COVID-19, unlock, emissions hotspots, AOD, air quality

## Abstract

The spread of the COVID-19 pandemic forced the administration to lock down in many countries globally to stop the spread. As the lockdown phase had only the emergency use of transportation and most of the industries were shut down, there was an apparent reduction in pollution. With the end of the lockdown period, pollution is returning to its regular emission in most places. Though the background was abnormally low in emissions (during the lockdown phase) and the reduced pollution changed the radiation balance in the northern hemispheric summer period, a modified pollution pattern is possible during the unlock phases of 2020. The present study analysed the unlock 1 and 2 stages (June–July) of the COVID-19 lockdown over India. The rainfall, surface temperature and cloud cover anomalies of 2020 for understanding the differences in pollutants variation were also analysed. The unlock phases show remarkable differences in trends and mean variations of pollutants over the Indian region compared to climatological variations. The results indicated changing high-emission regions over India to climatological variations and identified an AOD dipole with future emissions over India.

## 1. Introduction

The year 2020 had an exceptional COVID-19 spread, a pandemic claiming 806,543 deaths and 23,309,597 infections globally as of 23 August 2020 [1]. The pandemic has forced Governments in different parts of the world to lock down, which meant restricting movements, limited transportation, and the shutdown of industries and schools to prevent COVID-19 spread [2,3]. In addition to concerning health and economic issues during the lockdown [4,5,6], the COVID-19 lockdown resulted in a positive aspect of reducing pollution and reviving the environment globally [7,8]. Every major city around the globe witnessed a drastic reduction in pollutant concentrations during the pandemic spread period due to lockdown/restricted emission scenarios [9,10,11]. However, the magnitude of pollutant reduction varies spatially.

Various published manuscripts highlight the significant reductions in pollution during the COVID-19 lockdown globally. Although this work could not summarise all the findings, we summarised the findings by citing a few results from the published works to obtain a clear idea about air quality variations. Many works reported a significant reduction in various pollutants. Rodríguez-Urrego et al. [12] analysed Particulate matter with 2.5 micrometre or smaller diameter (PM_2.5_) reductions over fifty most polluted capital cities of the world, including countries from Asia, Europe, and American continents, during COVID-19 lockdown and found an overall averaged 12% decrease in PM_2.5_ concentrations over these cities. Collivignarelli et al. [13] reported a reduction of ~32–40% in PM_10_ (Particulate matter with 10 micrometre or smaller diameter) values, ~37–44% in PM_2.5_, ~49% in benzene, ~57.6% in CO, ~20% in SO_2_, ~47–51.4% in NO_2_ over the city of Milan, Italy. Otmani et al. [14] showed that PM_10_, SO_2_ and NO_2_ were reduced by 75%, 49% and 96% during the COVID-19 lockdown over Salé City (Morocco). Tobías et al. [15] reported that the black carbon and NO_2_ were reduced by 45 and 51%, while PM_10_ was reduced by 28 to 31% in Barcelona (Spain) during the COVID-19 lockdown. They also found that O_3_ levels increased from 33 to 57% for the eight hourly daily maxima during this period. Kerimray et al. [16] found that during the lockdown, the PM_2.5_, CO and NO_2_ concentrations were reduced by 21%, 49% and 35%, respectively, and O_3_ levels increased by 15% during the lockdown over Almaty, Kazakhstan. The enhanced O_3_ values due to reduced NO_X_ values were also reported by Sicard et al. [17]. They highlighted that Southern European cities (Nice, Rome, Valencia and Turin) have a different magnitude of variations in pollutants than Wuhan (China) during the lockdown, e.g., O_3_ increase (17% in Europe, 36% in Wuhan) and PM reduced ~42% in Wuhan and ~8% in Europe, with a substantial reduction (~56%) in NO_X_ in all cities. Zalakeviciute et al. [18] reported reduced concentrations of NO_2_ (−68%), SO_2_ (−48%), CO (−38%) and PM_2.5_ (−29%) in the capital city of Quito, Ecuador, during COVID-19 lockdown period. Broomandi et al. [19] found a decrease in SO_2_ by 5–28%, NO_2_ by 1–33%, CO by 5–41% and PM_10_ by 1.4–30%, respectively, over Tehran, Iran, during the COVID-19 lockdown period.

Pollution reduction is higher in major cities of developing countries such as India and China during the COVID-19 lockdown. Filonchyk et al. [20] found that CO and NO_2_ were reduced by 20% and 30%, while aerosol optical depth (AOD) and SO_2_ were significantly reduced over East China during the COVID-19 Lockdown period. Bao and Zhang [21] analysed PM_2.5_, PM_10_, SO_2_, NO_2_ and CO over 44 cities in northern China and found a significant decrease in levels of all pollutants during the COVID-19 lockdown. India’s megacities and state capitals reported a substantial pollution reduction during the COVID-19 lockdown period, e.g., [22,23]. Mahato et al. [24] found that PM_2.5_ and PM_10_ were reduced by >50%, NO_2_ by 52.68% and CO by 30.35% in Delhi, the capital of India, during the COVID-19 lockdown period. However, there are also reports of unperturbed air quality over specific zones over India during the COVID-19 lockdowns [25] related to power plant emissions. The main reason attributed to the reduced emissions of pollutants during the COVID-19 lockdown was the strict restriction on the movement of vehicles and the limited opening of industries [26]. The information is crucial and important to researchers, policy makers and the general public due to providing an unprecedented situation where in reality, one can measure the background pollution levels of different pollutants in different parts of the country [27,28].

Most global studies also reported a significant reduction in pollution during the COVID-19 lockdown period. Interestingly, a few studies report no decrease or increase in aerosol concentrations during the COVID-19 lockdown period. Nadzir et al. [29] found that PM_2.5_ and PM_10_ increased to 60% and 9.7% during the lockdown period over Kota Damansara, an urban area of Klang Valley, Malaysia, while at other sites, there are reductions by ~20 to 60%. The fossil fuel burnings in the residential area of Kota Damansara are the reason for the increased PM levels. Similar observations of elevated/maintained aerosol concentrations were reported by Ranjan et al. [30] over the central and eastern Indian region, where coal mines were in continuous operation during the lockdown period. Hari et al. [31] reported increased atmospheric methane over India during the COVID-19 lockdown. Apart from the pollution concentration changes during the COVID-19 lockdown, studies reported the changes in GNSS signals [32], crop-residual burning patterns [33] and the link of pollution patterns to COVID-19 spread [34] in India.

The Government of India limits the lockdown period to 31 May 2020 and states unlock phase on 1 June 2020 with limited movement and unlock two on 1 July 2020 with more flexibility to move (https://www.mygov.in/covid-19) (accessed on 15 August 2020). As unlock phases start, the transportation and industrial sectors regrow their emissions back to their routine, and pollution growth increases to higher levels in a couple of months. Even during the unlock phases, the public is taking precautions and avoiding unnecessary travel in India, keeping pollution growth under control. Though there has been vast reporting of pollution drop during the COVID-19 lockdown, published works did not extensively discuss the regrowth of pollution during the unlock phases in India. The present work analysed the pollution pattern change during the unlock phase 1 and 2 over India, compared the growth with the lockdown period of 2020 and understood the months of unlock/lockdown period of 2020 with climatological variation differences (2000–2019) for AOD, NO_2_ and SO_2_. We also analysed surface temperature, cloud cover, and rainfall anomalies for 2020 from the climatological means to understand the changes in the lockdown and unlock phases.

## 2. Materials and Methods

The area of study for the present work was India. The data on Aerosol Optical Depth (AOD) at 550 nm as a proxy for the aerosol load (PM pollution) were taken from the daily 1 × 1 degree resolution gridded level 3 collection 6.1 Dark Target Deep Blue (DT-DB) aerosol product of the Moderate Resolution Imaging Spectroradiometer (MODIS) on the Terra satellite [35] with an overpass at about 10:30 am local time. The product combines retrievals using the dark target algorithm [36,37] over water and the deep blue [38] algorithm over land and provides improved data coverage over both dark and bright surfaces [39,40]. The NO_2_ and SO_2_ daily data were obtained from the Ozone Monitoring Instrument (OMI) for April–July 2005 to 2020. For NO_2_, we used OMNO2d, cloud-screened total and tropospheric column Level-3 daily global gridded dataset with a spatial resolution of 0.25 × 0.25 degrees [41,42]. The SO_2_ datasets were analysed by OMSO2e data, which gives a total column density of SO_2_ in the planetary boundary layer. It is a Level-3 daily global gridded dataset with a spatial resolution of 0.25 × 0.25 degrees [43]. For rainfall, daily precipitation data from 2000 to 2020 were adopted from Global Precipitation Mission (GPM) Integrated Multi-satellite Retrievals (IMERG) version V06, which has a resolution of 0.1-degree × 0.1-degree. We also employed ERA-5 reanalysis data fields of surface temperature and cloud cover information (monthly data) for India from 2000 to 2020. ERA-5 is the fifth generation of European reanalysis datasets produced by the European Centre for Medium-Range Weather Forecast (ECMWF) [44]. In order to calculate mean variations, we averaged the data for any parameter over the study area. The industrial and power plant locations with annual coal consumption and emissions related to coal-fired power plants are adopted from Guttikunda and Jawahar [45].

In the present work, we attempted to identify the differences in pollutants during the lockdown and unlock phases of 2020. In order to observe the difference pattern without the COVID-19 lockdown scenario, we used the same months’ climatological variations. The work, therefore, is not commenting in any way that the COVID-19 lockdown or unlock phases changed the climatological patterns, but we are trying to understand the changes in 2020 with the help of climatological patterns.

## 3. Results

### 3.1. Understanding the Difference between Monsoon and Pre-Monsoon Variation

**Figure 1:**
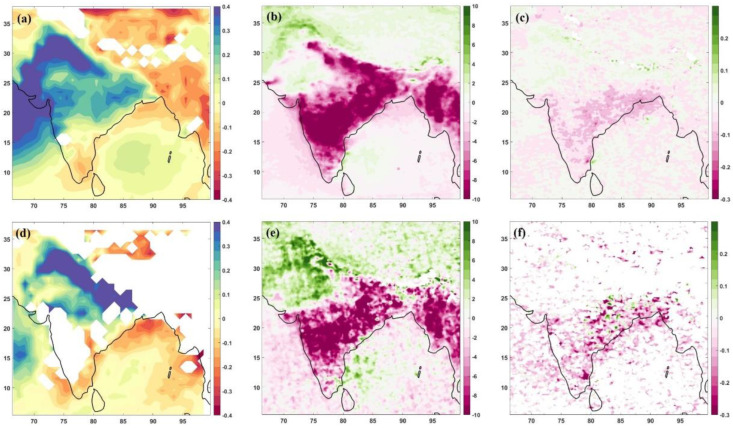
Difference between June–July and April–May values climatologically (upper row) and for 2020 (bottom row): AOD, left column: (**a**,**d**); NO_2_, middle column: (**b**,**e**); SO_2_, right column: (**c**,**f**). The AOD is dimensionless, NO_2_ is (×10^15^ molecules/cm^2^) and SO_2_ is in Dobson Units [DU].

As the unprecedented COVID-19 lockdown allows studying baseline emissions, it is interesting to learn how the unlock period changed the pollution pattern after reduced pollution levels over India. We computed the difference between monsoon months (June–July) and pre-monsoon months (April–May) for the climatological period, i.e., 2000–2019 for AOD and 2005–2019 for NO_2_ and SO_2_ (Figure 1a–c). The difference between unlock phase (June–July 2020) and lockdown phase (April–May 2020) for AOD, NO_2_, and SO_2_ was computed (Figure 1d–f) to see how the change in pollution growth of these pollutants concerns long-term average values over India.

The long-term average difference between monsoon and pre-monsoon months shows negative values in AOD (Figure 1a) over south-eastern coastal regions and positive values over the north, central and western areas of India. The positive values were higher as we moved northward side of India, as the rainfall is less during June–July over those regions, and the rainout/washout of AOD is the least. The variation in NO_2_ (Figure 1b) marks a decrease over India, including the Indo-Gangetic plains, except over a region encompassing the northern states of Haryana, Punjab, Himachal Pradesh, and parts of Gujrat and Rajasthan, where the values are either slightly positive or zero. The SO_2_ differences show positive values over the Indo-Gangetic plains, northern and western regions of India, and negative values over the rest of the Indian region (central, eastern, and southern parts). The results advocate that half of the country has higher values of SO_2_ during monsoon, and the other half has lower emissions during monsoon months compared to pre-monsoon months. The positive/negative values over the upper/lower part of the country are directly related to rainfall patterns from June to July [46].

The difference in pollutants from unlock to lockdown phase of 2020 has a different variation compared to the climatological mean in AOD, NO_2_ and SO_2_. The AOD differences (Figure 1d) cannot provide complete information over the whole of India due to data gaps. However, it is clear from the analysis that the high positive values of AOD, which were up to north India (Haryana, Punjab and western Uttar Pradesh), are now extended to the complete Indo-Gangetic plain during 2020. The results indicate that during the unlock phase, the AOD values were higher over the Indo-Gangetic plain, which was expected as the lockdown phase reduced pollution levels. The eastern and coastal regions of India have differences similar to climatological values, indicating that rainfall controls the variation from June to July. Similar to AOD, the NO_2_ variations (Figure 1e) also show higher values over the north and northwest sectors and lower values over the rest of India as climatological variations (Figure 1b). The higher values of NO_2_ over the north and the northwest region show a clear impact of lockdown reduced NO_2_ emissions in those regions due to reduced vehicular emissions, limited industrial activities, and controlled fossil fuel burning in household usage [44].

Interestingly, the rest of the country could not show such a marked difference from the unlock–lockdown period. The reasons may be continuous power plant operations and coal mining operations during the lockdown period (Ranjan et al., 2020). If we analyse SO_2_ variation during 2020 (Figure 1f), the higher values over the Indo-Gangetic plain no longer exist. The reduced values over the rest of the Indian region are continuing, but there are regions with higher values over eastern India (Odisha, West Bengal and Jharkhand). The variational changes in SO_2_ are reporting a reduction in SO_2_ emissions even during the unlock phase of 2020 over the Indo-Gangetic plain and higher emissions at places marked with industries and power-plants locations in eastern India. Though the results are not entirely unexpected, we needed to analyse the rainfall anomaly for the year 2020 for a better understanding of the differences discussed in the next section.

### 3.2. Rainfall Anomaly for the Year 2020

**Figure 2:**
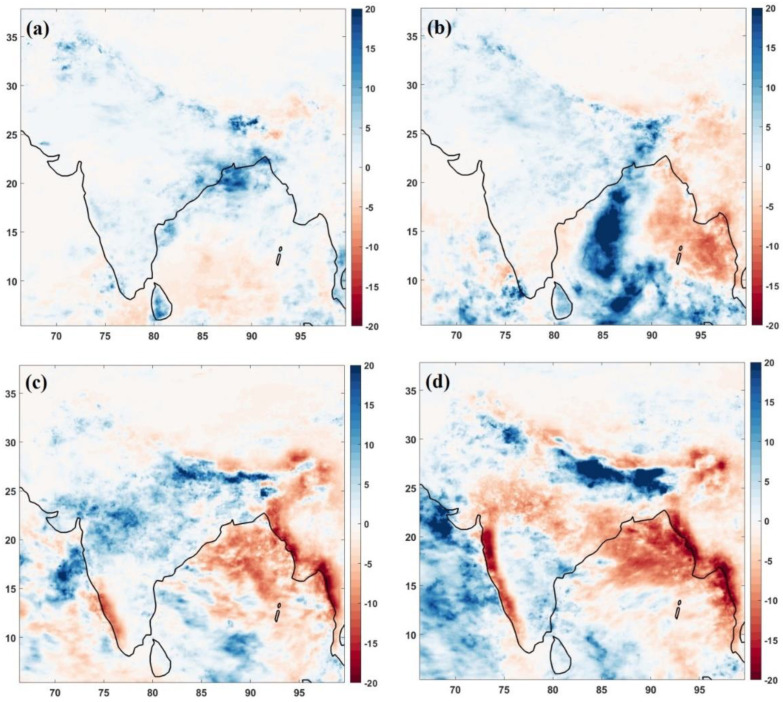
GPCC derived monthly rainfall anomaly of 2020 (from climatological mean of 2000–2019) for (**a**) April, (**b**) May, (**c**) June and (**d**) July. The units of rainfall are mm/month.

Figure 2 represents the rainfall anomaly for lockdown and unlock months of 2020 from the climatological mean of 2000–2019. For April (Figure 2a), the rainfall increased for the Indo-Gangetic plain and coastal belt from south to east, especially over the eastern Indian region (Odisha and West Bengal). Though there is no decrease in rainfall all over India, central India did not show any significant change in rainfall amount for April. May 2020 also marked higher rainfall (Figure 2b) over the Indian region. This month, the passage of supercyclone Amphan marked higher rainfall in the affected areas. The monsoon onset month of June (Figure 2c) had a different rainfall pattern in 2020 compared to April and May. The southwest coastal regions of India (Kerala and Karnataka) had deficit rainfall, while other southern states (Tamilnadu, Andhra Pradesh and Telangana) did not significantly change rainfall for the month. Higher precipitation was observed for the western part (Maharashtra and Gujrat), central India (Chhattisgarh, Madhya Pradesh and Rajasthan), eastern India (Odisha, West Bengal and Jharkhand) and parts of Indo Gangetic Plain (Uttar Pradesh and Bihar). The values were higher than April and May variations. For July 2020 (Figure 2d), the Indo Gangetic plain region (parts of Uttar Pradesh and Bihar) and northern and central India (Haryana, Punjab and Rajasthan) received higher rainfall amounts. Central India (Madhya Pradesh, Chhattisgarh) and the west coast of India (including regions of Maharashtra, Karnataka and Kerala) showed a significant decrease in rainfall for July 2020. When we combine the difference between monsoon and pre-monsoon months, there is a reduction in rainfall amount over the southwest coast of India and an increase in rainfall over the Indo-Gangetic plains.

### 3.3. AOD, NO_2_ and SO_2_ Anomalies for Lockdown and Unlock Phases of the Year 2020

**Figure 3:**
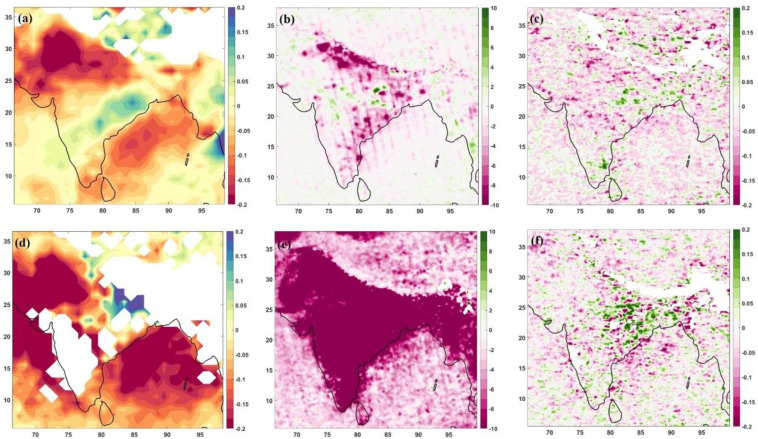
Anomaly of April–May (top row) and June–July (bottom row) from 2020 observations: AOD, left column: (**a**,**d**); NO_2_, middle column: (**b**,**e**); SO_2_, right column: (**c**,**f**). The AOD is dimensionless, NO_2_ is (×10^15^ molecules/cm^2^) and SO_2_ is in Dobson Units [DU].

Figure 3 shows the anomaly of the year 2020 from the climatology for the lockdown (April–May) and unlock phase (June–July) over the Indian region. The climatology was made only for April–May and June–July months (separately) to analyse these variations with respect to lockdown and unlock phases. One expects reduced pollutants in the lockdown phase and increased values during the unlock phases. However, the results are not as expected for unlock stages. The AOD values in the lockdown phase (Figure 3a) show a significant reduction over north-west regions (Haryana, Punjab, Himachal Pradesh, Delhi, Jammu and Kashmir, and parts of Uttar Pradesh). However, the eastern states of Odisha, West Bengal and Jharkhand show an increase in the values for the lockdown period. For the monsoon season, we had more missing data in AOD. Figure 3d indicates a higher decrease in the regions of the lockdown phase in addition to central and western Indian areas (Madhya Pradesh, Chhattisgarh, Gujrat and Maharashtra), where the increase over Odisha, West Bengal and Jharkhand was higher than that of lockdown phase, observed in Figure 3a. 

The NO_2_ variations over India during the lockdown phase (Figure 3b) show a decrease over the Indo-Gangetic plains and southern states of Tamilnadu, Andhra Pradesh and Telangana. At the same time, some hotspot regions were observable over eastern India (Odisha, West Bengal, and Jharkhand). When we checked the monsoon months (unlock phases), there was a uniform decrease in NO_2_ of ~10 µg/m^3^ per day over India (Figure 3e). No hotspot was visible for the unlock phase in the NO_2_ anomaly variations. As in the unlock phases, the movement started, and with usual traffic and industries, the NO_2_ concentrations were supposed to return to typical values in 2020; this result was unexpected. The low concentrations indicate that the emissions were restricted even in the unlock phases. India uniformly shows reduced emissions, which was not even the case during the lockdown period. The uniform decrease during the unlock phases may also be attributed to higher rainfall received during 2020 (Figure 2c,d), adding the rainout and washout of NO_2_ in higher amounts.

The SO_2_ variations, though, are trivial to understand for the lockdown and unlock phases of 2020, as they can be connected to coal burning [47,48]. Unlike AOD or NO_2_ variations, SO_2_ anomalies during lockdown months (Figure 3c) show higher emissions at locations of coal-fired power plants in eastern regions (West Bengal, Odisha, and Jharkhand), south (Chennai) and a few places in north India. Other than these identified locations, the SO_2_ concentrations showed a reduction over the whole of India. For the unlock months (Figure 3f), the eastern India region emerged as a higher emission region. West Bengal, Odisha and Jharkhand are the states with hotspot regions for SO_2_ emission. The combined discharge from the mineral industries, mines and coal-fired power plants keeps the higher emissions of SO_2_ over the area compared to the rest of the country. Even with higher rainfall (Figure 2) and a lockdown/unlock situation in place, the year 2020 marked higher SO_2_ emissions over the eastern region from climatology, indicating the rise in production and capacity of various industrial establishments over the area [45].

### 3.4. Relationships between Meteorological and Air Quality Factors

The air pollutants variation during the COVID-19 lockdown and unlock phases were also impacted by the meteorological conditions during this period. Most of the studies explored the change in pollutants/emissions during the lockdown well, but the exploration of meteorological changes and their feedback process is less explored during these phases. The relationship between meteorological conditions and pollution changes was explored globally and found to have a feedback process between the two [49]. For the COVID-19 lockdown period, Singh et al. [26] explored the surface temperature, wind speed and rainfall along with pollutants. They found that the existence of anticyclonic motion over the Bay of Bengal and the Arabian Sea, along with prevailing north westerlies, is one prominent feature observed during the lockdown years of 2020. The study reported that all the meteorological parameters follow a similar spatial variation, which was also confirmed by other studies, e.g., [22,50,51,52]. The reduced pollutant concentration also acted as a feedback process of impacting the meteorological parameters, and Pal et al. [53] reported a significant reduction in regional temperature related to emissions over India during the COVID-19 period. Moreover, the boundary layer height was ~70% higher, with a 40–60% increase in relative humidity and 20–40% slower wind speeds (on a synoptic scale) during the COVID-19 lockdown [54]; further making the available concentrations of pollutants to appear low.

**Figure 4:**
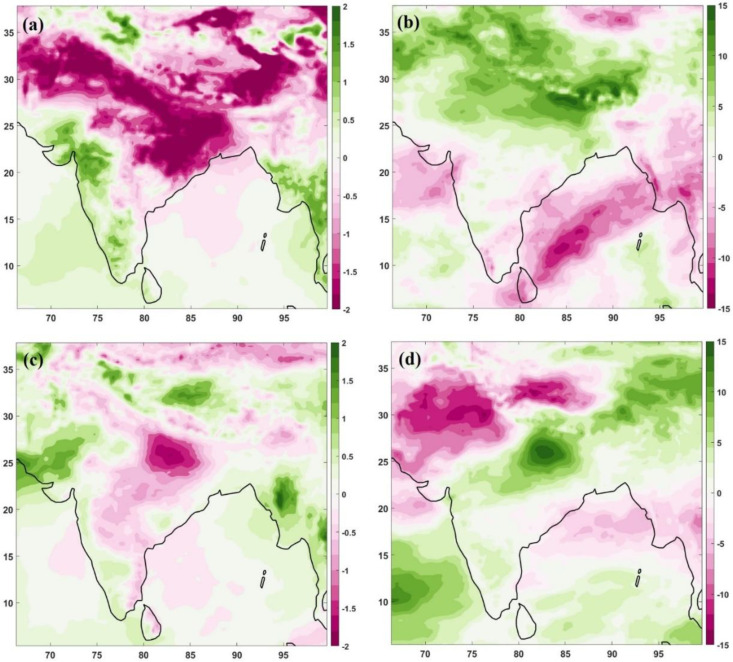
Anomaly of April–May (top row) and June–July (bottom row) from 2020 observations: surface temperature, left column: (**a**,**c**); cloud cover, right column: (**b**,**d**). Temperature is in K, and cloud cover is in percentage (%).

In order to further explore the relationship between the meteorological factors and pollutant concentrations, we analysed the surface temperature and cloud cover variations over the Indian region. The variations in surface temperature and cloud cover were explored for the same period as the pollutants for April–May and June–July. We utilised ERA5 reanalysis data to analyse the surface temperature and cloud cover over the Indian region, and Figure 4 depicts the anomalies of 2020 from the 2000–2019 values.

The lockdown phase temperature anomaly (Figure 4a) shows that a large part of India (including north India, Indo-Gangetic plains, and eastern India) has a negative temperature anomaly during the lockdown phase. However, the western and southern regions had positive anomaly values during the lockdown phase. During the lockdown phase, the cloud cover significantly increased over India, and the northern and eastern parts (including the Indo-Gangetic plains) showed up to a 15% increase in the cloud cover (Figure 4b). However, the central, western, and southern parts did not show any such increase in cloud cover during the lockdown; instead, there was a slight decrease in cloud cover values.

The temperature still shows a negative anomaly for the unlock phases over most of India. However, the magnitudes for a more prominent part are reduced, except over Bihar. The higher positive magnitude of anomaly over western India also shows a shift northward, i.e., over the adjoining Pakistan region in the unlock phase (Figure 4c). The cloud cover anomaly also has negative values over the northern part for unlock phases (Figure 4d). However, the state of Bihar in north India still shows a positive anomaly. This creates a dipole pattern in the cloud cover over north India, where both positive and negative cloud cover anomalies are observed. The surface temperature and cloud cover anomalies show an inverse relationship (Figure 4). The pre-monsoon negative temperature anomalies are over the regions of positive cloud cover anomalies during the lockdown period (Figure 4a,b). Similarly, the negative temperature anomalies during the unlock phase agree with positive cloud cover anomalies during the unlock phase (Figure 4c,d).

## 4. Discussion

The present study investigated the patterns of AOD, NO_2_ and SO_2_ during the lockdown and unlock phases of COVID-19 over India. The study incorporated the rainfall, surface temperature and cloud cover patterns for the same periods to understand better how the feedback between these meteorological variables and pollution evolved during the study period. The unique way of pollutant variation during the COVID-19 lockdown reduced pollution on average for the global scenario. The changes from lockdown to unlock phases differ from the previous emission patterns, where the pollution concentrations were relatively higher.

For the unlock phase, the pollution concentrations were not as high as they used to be in the climatology (Figure 1). However, the Indo-Gangetic plain emerged as the region of reduced aerosol pollution during the lockdown phase. When we analysed the variation in NO_2_ and SO_2_, the difference between unlock and lockdown phase indicated that the levels in unlock phases are showing reduced concentrations compared to the lockdown phase over India, except in parts of northern and central India, which mark the impact of lockdown over NO_2_ and SO_2_, with substantial reductions in concentrations. The precipitation anomalies (Figure 2) during the lockdown period show positive values over India. In contrast, during the unlock phases, the values are primarily positive over India in June. July offered a negative anomaly over central and southwest India, but there were increased (positive) anomalies over the Indo-Gangetic plain.

The unlock and lockdown phase anomalies from climatology (Figure 3) indicated that though most of India observed reduced pollution concentrations during the lockdown phase, the hotspots are visible over eastern India. However, more interestingly, the unlock phases show reduced pollution from climatological values for AOD and NO_2_ for India. For SO_2_, the country shares a positive–negative anomaly map, indicating that the values increased/decreased over places for the country during the 2020 unlock period from the climatological values. The AOD dipole pattern over the Indian region [50] is evident in both pre-monsoon (lockdown) and monsoon (unlock) sub-sections.

We analysed India’s surface temperature and cloud cover values (Figure 4) to find if the temperature and cloud cover feedback works with the pollutant variations over the country and attempt to identify the feedback mechanism. The surface temperature anomalies for unlock and lockdown phase from 2020 show a reduced temperature over the north, east and central India, with higher magnitudes during the lockdown period. The higher temperature regions were also observed over western India during the lockdown and unlock phases. Cloud cover showed an inverse relation to surface temperature, and the northern and eastern areas showed an increase in cloud cover during the lockdown phase. However, during the unlock phase, there was a significant reduction in cloud cover over the northern region. However, the eastern part continued to have higher cloud cover, which created a dipole structure in cloud cover. The temperature anomalies show a good agreement with AOD during the lockdown and unlock phases. The reduction/increase in temperature is related to the reduction/increase in AOD over northern, western and southern regions of India. However, the increased AOD over eastern India is not in agreement with reduced temperature during the lockdown. The temperature pattern changes are also in agreement with NO_2_ variations during the lockdown phase, but the unlock phase is unable to explain the temperature dipole pattern over India. SO_2_ concentrations are poorly correlated with temperature variations over India and not matching either with lockdown or unlock phases. During the unlock phase, India showed a negative anomaly in NO_2_ during 2020 from the climatological values, with negative temperature and positive cloud cover anomaly values. One can also argue that as the lockdown was unprecedented and the reductions were primarily due to the absence of emission sources, one may not obtain the feedback mechanism between the temperature and cloud cover with the pollutants, which is the case in the present analysis. However, there may be feedback from pollution to meteorology, and the reduced temperature pattern during the unlock and lockdown phase can reduce the country’s AOD, NO_2_ and SO_2_ values.

## 5. Conclusions

The lockdown and unlock phases related to COVID-19 in 2020 gave us a unique opportunity to understand the base levels of AOD, NO_2_ and SO_2_ and their sector-wise growth in India. The results show changed emission patterns during the COVID-19 times and significantly lower values during monsoon months (unlock phases 1 and 2) compared to climatological variations. We summarised the findings of the present work as follows:

- The difference between monsoon and pre-monsoon months climatology revealed that the north region has higher AOD values. During the COVID-19 unlock/lockdown phases, the extent of the higher AOD region extended to the total Indo-Gangetic plain. The AOD dipole existed during the anomaly of the lockdown and unlock stages of 2020. However, NO_2_ variations were lower for the whole of India during unlock months and not for the lockdown period. The increased rainfall amounts in 2020 may be the reason. The SO_2_ variations show hotspot emission regions over eastern India during both the lockdown and unlock phases compared to climatological variations;
- The NO_2_ reduction during monsoon months to pre-monsoon months was evident in the whole of India except north region (Haryana, Punjab, Delhi, Himachal Pradesh, Rajasthan, Uttarakhand and parts of Uttar Pradesh) for climatological variations. However, during the COVID-19 times of 2020, the unlock months show a positive change (increase in values) over these states, which is different from the climatological variation. The SO_2_ variations, however, move in line with climatological variations during 2020, except for higher emission SO_2_ sites identified during the unlock–lockdown phase over Odisha, West Bengal and Jharkhand;
- The COVID-19 lockdown and unlock months received higher rainfall than climatological variations over India. The surface temperature anomalies show reduced temperature for lockdown and unlock phases, more prominently during the lockdown. During the lockdown phase, there was an increase in cloud cover over northern and eastern India. In contrast, the unlock phases show a dipole pattern—a decrease over the northwestern part and an increase over east India.

The results indicated that during the unlock phase 1 (June–July 2020), the variations in AOD, NO_2_ and SO_2_ were smaller than climatological values due to reduced emissions associated with rainout and washout effects. There is a need to conduct a more comprehensive analysis to understand the reasons for such variations over different regions of India, particularly with surface temperature, radiation, and cloud cover during the study period.

## Data Availability

Satellite data analysed for the study are freely accessible from https://earthdata.nasa.gov/ (accessed on 1 March 2022). ERA5 datasets used in the study were obtained from https://cds.climate.copernicus.eu/cdsapp#!/dataset/reanalysis-era5-land?tab=form (accessed on 24 July 2022).
